# Fungal primary and opportunistic pathogens: an ecological perspective

**DOI:** 10.1093/femsre/fuae022

**Published:** 2024-08-08

**Authors:** Sybren de Hoog, Chao Tang, Xin Zhou, Bruna Jacomel, Bruno Lustosa, Yinggai Song, Hazal Kandemir, Sarah A Ahmed, Shaoqin Zhou, Ricardo Belmonte-Lopes, Yu Quan, Peiying Feng, Vania A Vicente, Yingqian Kang

**Affiliations:** RadboudUMC-CWZ Centre of Expertise for Mycology, 6525GA Nijmegen, The Netherlands; Foundation Atlas of Clinical Fungi, 1214GP Hilversum, The Netherlands; Key Laboratory of Environmental Pollution Monitoring and Disease Control, Ministry of Education of Guizhou & Key Laboratory of Medical Microbiology and Parasitology, School of Basic Medical Sciences, Guizhou Medical University, 561113 Guiyang, China; Postgraduate Program in Microbiology, Parasitology and Pathology, Biological Sciences, Department of Basic Pathology, Federal University of Paraná, 81531-980 Curitiba, Brazil; Department of Medical Microbiology, Radboud University of Nijmegen, 6525AJ Nijmegen, The Netherlands; RadboudUMC-CWZ Centre of Expertise for Mycology, 6525GA Nijmegen, The Netherlands; Key Laboratory of Environmental Pollution Monitoring and Disease Control, Ministry of Education of Guizhou & Key Laboratory of Medical Microbiology and Parasitology, School of Basic Medical Sciences, Guizhou Medical University, 561113 Guiyang, China; RadboudUMC-CWZ Centre of Expertise for Mycology, 6525GA Nijmegen, The Netherlands; Third Affiliated Hospital of Sun Yat-sen University, 510630 Guangzhou, China; Postgraduate Program in Microbiology, Parasitology and Pathology, Biological Sciences, Department of Basic Pathology, Federal University of Paraná, 81531-980 Curitiba, Brazil; Canisius Wilhelmina Hospital, 6532SZ Nijmegen, The Netherlands; RadboudUMC-CWZ Centre of Expertise for Mycology, 6525GA Nijmegen, The Netherlands; Postgraduate Program in Engineering Bioprocess and Biotechnology, Department of Bioprocess Engineering and Biotechnology, Federal University of Paraná, 81531-980 Curitiba, Brazil; Department of Dermatology and Venerology, Peking University First Hospital,100034 Beijing, China; Westerdijk Fungal Biodiversity Center, 3584CT Utrecht, The Netherlands; RadboudUMC-CWZ Centre of Expertise for Mycology, 6525GA Nijmegen, The Netherlands; Foundation Atlas of Clinical Fungi, 1214GP Hilversum, The Netherlands; RadboudUMC-CWZ Centre of Expertise for Mycology, 6525GA Nijmegen, The Netherlands; Key Laboratory of Environmental Pollution Monitoring and Disease Control, Ministry of Education of Guizhou & Key Laboratory of Medical Microbiology and Parasitology, School of Basic Medical Sciences, Guizhou Medical University, 561113 Guiyang, China; RadboudUMC-CWZ Centre of Expertise for Mycology, 6525GA Nijmegen, The Netherlands; Postgraduate Program in Microbiology, Parasitology and Pathology, Biological Sciences, Department of Basic Pathology, Federal University of Paraná, 81531-980 Curitiba, Brazil; RadboudUMC-CWZ Centre of Expertise for Mycology, 6525GA Nijmegen, The Netherlands; Foundation Atlas of Clinical Fungi, 1214GP Hilversum, The Netherlands; Third Affiliated Hospital of Sun Yat-sen University, 510630 Guangzhou, China; Postgraduate Program in Microbiology, Parasitology and Pathology, Biological Sciences, Department of Basic Pathology, Federal University of Paraná, 81531-980 Curitiba, Brazil; Postgraduate Program in Engineering Bioprocess and Biotechnology, Department of Bioprocess Engineering and Biotechnology, Federal University of Paraná, 81531-980 Curitiba, Brazil; Key Laboratory of Environmental Pollution Monitoring and Disease Control, Ministry of Education of Guizhou & Key Laboratory of Medical Microbiology and Parasitology, School of Basic Medical Sciences, Guizhou Medical University, 561113 Guiyang, China

**Keywords:** clinical fungi, pathogenicity, opportunism, adaptation, life cycle, infection kinetics

## Abstract

Fungal primary pathogenicity on vertebrates is here described as a deliberate strategy where the host plays a role in increasing the species’ fitness. Opportunism is defined as the coincidental survival of an individual strain in host tissue using properties that are designed for life in an entirely different habitat. In that case, the host's infection control is largely based on innate immunity, and the etiologic agent is not transmitted after infection, and thus fungal evolution is not possible. Primary pathogens encompass two types, depending on their mode of transmission. Environmental pathogens have a double life cycle, and tend to become enzootic, adapted to a preferred host in a particular habitat. In contrast, pathogens that have a host-to-host transmission pattern are prone to shift to a neighboring, immunologically naive host, potentially leading to epidemics. Beyond these prototypical life cycles, some environmental fungi are able to make large leaps between dissimilar hosts/habitats, probably due to the similarity of key factors enabling survival in an entirely different niche, and thus allowing a change from opportunistic to primary pathogenicity. Mostly, such factors seem to be associated with extremotolerance.

## Teaser

The authors conclude that for a primary pathogenic species, the infection is strategic, while ecological strategies of opportunistic pathogens do not include vertebrates, and the infection is detrimental even if the infecting fungus survives.

## Introduction and definitions

The fungi comprise one of the species-rich branches in the Tree of Life. Since the COVID-19 outbreak in 2021, which regionally led to severe fungus-associated infections (Ghazi et al. 2021), the general public is aware of the fact that microbes may pose a significant health problem unexpectedly, and among these are fungi. In nearly 160 years of medical mycology research, over 800 fungal species have been proven to be able to infect humans and other vertebrate animals (de Hoog et al. 2020). This seems like a high number, but compared to the millions of existing species (Hawksworth and Lucking 2017 suggested 3.8 million), this would amount to only 0.0002%. Although novel infectious species are still being identified at a regular pace, the ability to infect an animal host remains an extremely rare fungal property.

Infection is usually understood as host damage from the perspective of human health, but from the perspective of the pathogenic fungus, *Homo sapiens* is rarely the preferred host. Pathogenicity is a relative feature (Rokas 2022); the great majority of fungi with infecting ability have their natural habitat in association with non-human vertebrates and behave non-typical in case of infection. Additionally, the invasive properties and course of the disease are also dependent on a multitude of host factors and on the portal of entry of the agent. The degree of host damage is a component of fungal virulence interplaying with host factors; pathogenicity, i.e. the evolutionarily determined course of events in the host, is relative to the species’ natural life cycle.

For an ecological definition of vertebrate pathogenicity, we distinguish those fungi where infection is advantageous to the species’ survival, from those where infection can be considered as detrimental. In the Darwinian sense, this **primary pathogenicity** is an intrinsic property of the fungus, designed to increase the species’ fitness. Use of the preferred vertebrate host anywhere in the natural life cycle enhances progeny and dispersal. However, even when this host is essential for the species, it is indispensable for each individual only when the fungus is transmitted from host to host (host-transmitted pathogens, e.g. *Trichophyton concentricum*; Table 1), and much less so in the pathogens with a double life cycle (environmental pathogens, e.g. *Coccidioides immitis*; Table 1), returning to the environment after infection (Carpouron et al. 2022). The advantage of infection for the environmental pathogens is underlined by their production of specialized forms in animal tissue, as observed in the dimorphic pathogens in *Ajellomycetaceae* (*Blastomyces, Histoplasma*, e.a.): the tissue phases are more pronounced in species that are more prevalent in infection (Jiang et al. 2018). When residence in animal tissue is part of the natural life cycle, the fungus must be able to escape from the host after infection for survival and dispersal—although this hypothesis has not been proven in most cases. Transmission is theoretically regarded as a prime criterion of pathogenicity. Note that damage to the host may vary from severe to absent and is therefore not included in the definition.

**Table 1.** Definitions used in this paper.

**Symbiont**, fungus having continuous or temporary association with a vertebrate host as its natural lifestyle, covering the entire spectrum from primary pathogenicity to commensalism.
**Virulence**, degree of host damage upon infection.
**Pathogenicity**, evolutionarily determined course of events in the host, *Ceteris paribus*.
**Pathogen**, fungus able to cause disease in vertebrate hosts.
**Primary pathogen**, fungus using growth in vertebrate tissue to enhance its survival and/or dispersal in any stage in its life cycle, using a specialized tissue phase if systemic or subcutaneous and being having the ability to escape from the host. Examples: *Histoplasma capsulatum* with tissue phase and *Trichophyton concentricum* with cutaneous transmission.
**Opportunistic pathogen** (syn.: secondary pathogen), fungus coincidentally able to survive in vertebrate tissue due to factors that are applied in its natural habitat and able to grow in vertebrate tissue only upon damage of the host functionalities due to e.g. immune or metabolic disorders, wounds, or medication. Example: *Rhizopus arrhizus*.
**Facultative pathogen**, fungus for which the host is only one of the niches where it is able to reproduce.
**Obligate pathogen**, primary pathogen unable to produce assimilative thallus outside the host. Example: *Batrachochytrium dendrobatidis*.
**Commensal**, fungus using growth on or in the vertebrate body enhancing survival and/or dispersal in any stage in its life cycle without tissue invasion in the immunocompetent host. Examples: *Malassezia furfur* and *Candida albicans*.
**Environmental pathogen**, primary pathogen transmitted with an environmental phase after infection, the host not being contagious. Example: *Coccidioides immitis*.
**Double life cycle**, irregularly intermittent growth of an environmental pathogen in a vertebrate host and in its environmental habitat. Example: *H. capsulatum*.
**Host-transmitted pathogen**, primary pathogen with host-to-host transmission, the host being contagious. Example: *T. concentricum*.
**Saprobe**, fungus feeding from non- vertebrate organic debris in the environment. Example: *Aspergillus fumigatus*.
**Preferred host**, healthy vertebrate species where the fungus is found under its natural conditions and where it exhibits its lowest degree of virulence. Example: rodent-associated *Emmonsia crescens*.
**Lifestyle**, basic way of feeding of a fungus.
**Strategy**, factors promoting the preferred lifestyle of the fungus and enhancing maximum progeny, potentially evolve towards higher degree of adaptation.
**Fitness**, the relative reproductive success of an individual or genotype.
**Acquired Immunodeficiency Syndrome-defining fungus**, fungal infection in an early stage of development of AIDS. Example: *Talaromyces marneffei*.
**Enzootic**, animal disease in a particular geographic area. Example: *T. marneffei* on bamboo rats.

In contrast, an opportunistic fungus has a preferred habitat outside the vertebrate body. We define an **opportunistic pathogen** (or secondary pathogen) here as a fungus where infection is not part of its natural life cycle (Table 1). It may have properties needed for survival in its natural habitat, which coincidentally also enhance resistance to phagocytosis, exemplified by extremophilic, surface-colonizing fungi that contain melanin (Gostincar et al. 2018), a component required for mitigation of effects of irradiation (Cordero and Casadevall 2017) and also promoting resistance to phagocytosis (Liu et al. 2014). When inoculated into an animal, the individual isolate aims to survive, but even when successful and overcoming the host's immune response, residence in the host is detrimental to the species as it diminishes progeny and fitness. When the propagule would have landed in a preferred habitat, it could have produced a much higher offspring. Such infections are often chronic, and the etiologic agent is likely to die with the host, and thus the infecting individual is lost for the fungal population.

Given the above fundamental difference between primary pathogenicity and opportunism, we may conclude that for the former the infection is strategic, while ecological strategies of opportunistic species do not include vertebrates, and the infection is detrimental even if the infecting fungus survives. For opportunists, the individual benefit is not in line with the benefit for the species. This distinction is not always unambiguous, as numerous pathogens infect non-optimal hosts, and then behave in a more opportunistic fashion.

### Examples of primary pathogenic fungi

One of the best examples of a pathogenic fungus is *Batrachochytrium dendrobatidis*, the Chytrid fungus capable of infecting a large number of frog species all over the globe (Castro Monzon et al. 2020) and a major driver of frog decline (Skerratt et al. 2007). Pathogens living in association with their preferred host are likely able to infect healthy individuals and provoke a defined course of disease, which is usually mild. However, severe infection and host decline are usually observed in hosts that are immunologically naïve to the fungus, while pathogens mostly remain innocuous when living in prolonged association with their preferred host. The balance is restored in the aftermath of the epidemic, leading to the co-existence of host and low-virulence pathogen (Hollanders et al. 2023) and co-evolution of resistant hosts (Mutnale et al. 2018). Similar observations were made with *Pseudogymnoascus destructans*, the fungus causing devastating white-nose disease in bats in the United States, while the same fungal lineage resided in Europe without causing significant disease (Zukal et al. 2016). This suggests that a close relationship between pathogen and host requires adaptation to increasing resistance and decreasing virulence to a particular optimum, the transmission-virulence trade-off (Kun et al. 2023). Host-to-host transmitted pathogens, such as anthropophilic dermatophytes, have their assimilative thallus in host tissue, and thus need the host not only for distribution and survival, but also for growth. Due to their dependence on the animal, there is more evolutionary pressure towards the decrease of virulence to reestablish coexistence. As an example, the most successful dermatophyte, *Trichophyton rubrum*, often causes nearly asymptomatic tinea pedis. The ultimate host-pathogen interaction equilibrium (Kirchner and Roy 2002) needs to be reached before the host is driven to extinction. In line with this, the dimorphic pathogens *Histoplasma capsulatum* and *C. immitis* mainly cause infection in healthy individuals entering endemic foci when they are immunologically naïve. Virulence, i.e. the degree of damage to the host, differs widely between pathogenic species. Extinction of the host leads to extinction of the pathogen, and therefore this can be regarded as an inefficient, primitive strategy. In adapted fungi such as *Emmonsia crescens* commonly causing adiaspiromycosis, the rodent or armadillo hosts (Borman et al. 2018, Hughes and Borman 2018, Navas-Suarez et al. 2021) seem to experience limited harm, which supports the hypothesis that mitigation of virulence increases the species’ survival in the long run. Similarly, *Talaromyces marneffei* in bamboo rats (Cao et al. 2011) and *H. capsulatum* in bats (Gugnani and Denning 2023) are likely to be long-established environmental pathogens. This particularly holds true for the latter species, which has accumulated enormous diversity in anonymous markers over time (Rodrigues et al. 2020).

**Figure 1.**
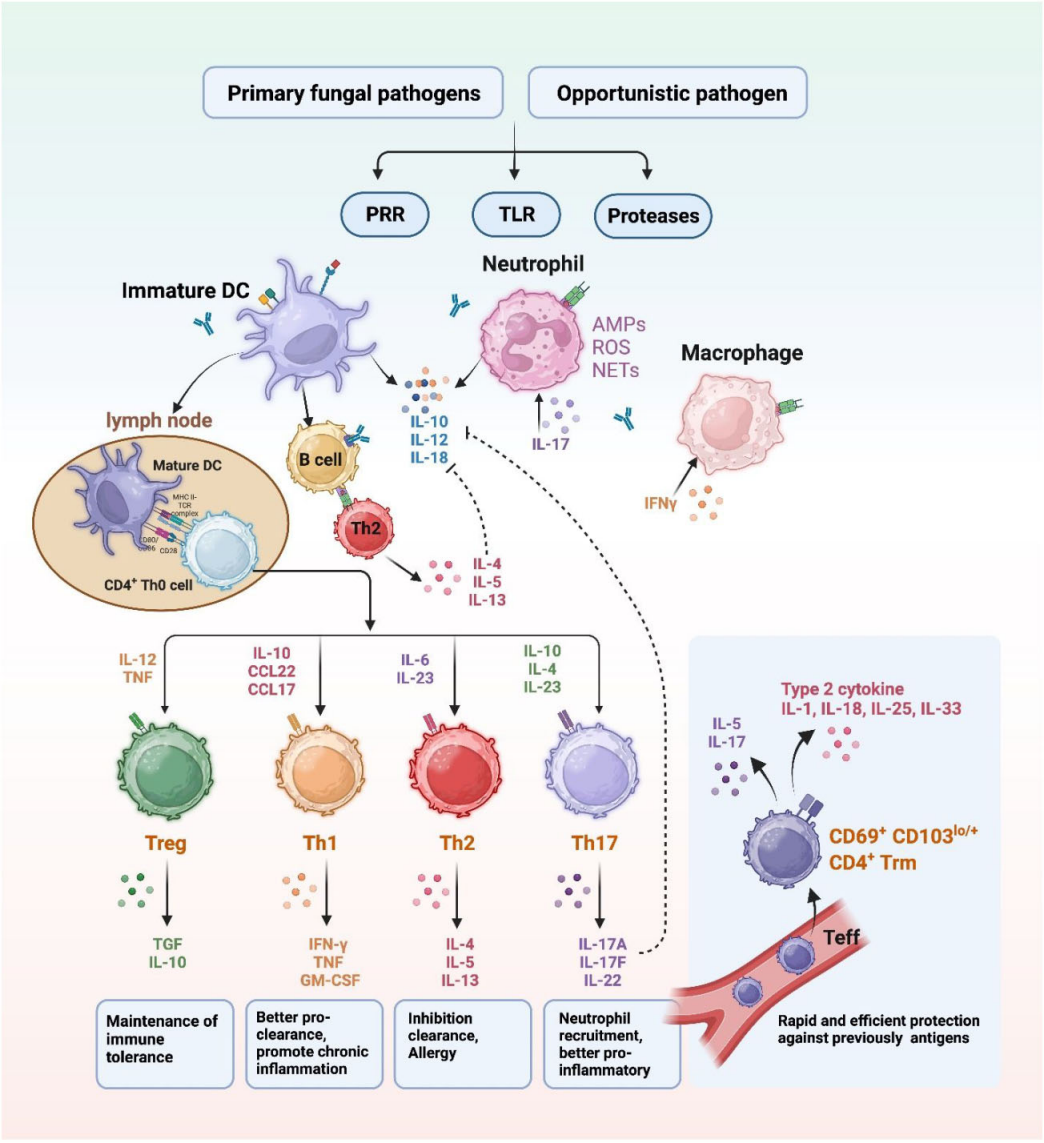
Summary of immune response against primary and opportunistic pathogens, with innate and adaptive arms.

Different from the mechanism of *Batrachochytrium*, host-to-host transmission is very rare among fungi. Primary pathogenic as well as opportunistic fungi are mostly acquired from sources outside the host species, i.e. the environment, and in the case of humans often other mammals. In humans, contagious fungal infections are limited to the anthropophilic dermatophytes. The anthropophilic dermatophytes have become extremely successful globally (Havlickova et al. 2008) by transmission via skin flakes loaded with fungal cells, but from an evolutionary point of view, this is a dead end. Sexuality is lost because the large, elaborate fruitbody can only be produced in the environment, as is characteristic for geophilic *Arthroderma* species which just use terrestrial animals for dispersal via fur colonization. Host shifts to anthropophily have taken place repeatedly from domesticated animals since the early days of animal husbandry. Four out of six anthropophilic dermatophytes have an identifiable, closely related zoophilic counterpart (Zhan et al. 2018). The process of dermatophyte adaptation has been described in the *Microsporum canis* complex (Zhou et al. 2023), a well-delimited group of species containing a species on cats (*M. canis*) and one restricted to humans (*Microsporum ferrugineum*) (Fig. 1). Cat–human infection by the original zoophilic species is common, often leading to inflammatory tinea capitis on the scalp. Transmission from an infected human requires lower virulence and less inflammation, which is beneficial for the fungus, and thus can be regarded as a new strategy. The preferred host of *M. canis* is the cat, whereas that of *M. ferrugineum* is the human. The anthropophilic dermatophyte *T. rubrum* may have evolved at an earlier stage towards its nearly commensal lifestyle, with no direct ancestral zoophile being identifiable.

Interestingly, *H. sapiens* is probably the only vertebrate carrying a large number of host-specific dermatophytes. The response to hairs of animal fur did not provoke significant differences in a panel of species of dermatophytes (Tang et al. 2023), but a transition from fur colonization to immunologically active naked skin, even though both environments share keratin being the essential nutritional source of dermatophytes, is a significant step. With domestication, mankind has created its own adapted pathogens (Tang et al. 2021), and this suggests that the dermatophyte host shifts take place within a relatively short timeframe (Tang et al. 2022). Tinea capitis is likely to be an initial phase in the host shift (Kandemir et al. 2020, Zhou et al. 2023), but this often highly inflammatory type of infection obviously is inefficient for host-to-host transmission, and further adaptation is required.

*Batrachochytrium dendrobatidis*, the frog pathogen of which the environmental part of the life cycle consists of just waterborne zoospores, occurs on a wide variety of frog species (Mutnale et al. 2018). Other fungi have been described with a very wide ecological amplitude. Sigler (2005) reported *E. crescens* from a large number of small animal species, but rarely on larger hosts; the terrestrial rodents are likely to be preferred hosts (Fig. 1; Borman et al. 2009). Some close relatives of *Emmonsia* in *Emergomyces* have thus far mainly been found in humans (Jiang et al. 2018, Friedman and Schwartz 2019), and seem to have a narrow amplitude with host restriction. Environmental pathogens can also be restricted by factors in their environmental habitat, such as *Coccidioides* species in desert soil (Fisher et al. 2002). Other enzootic and endemic fungi can be limited in their expansion by factors such as the distribution of the preferred host (Hrycyk et al. 2018) or climate (Gorris et al. 2019).

Frequently mentioned factors promoting infection are (i) adhesion (Hogan et al. 1996), (ii) thermotolerance (Firacative 2020), (iii) tissue lysis (Kohler et al. 2014), (iv) melanin formation (Smith and Casadevall 2019), (v) toxin production (Brown et al. 2021), (vi) protective and biofilm capsule formation (Morse et al. 2019), (vii) production of hydrolytic and proteolytic enzymes (Schaller et al. 2005), and (vii) dimorphism and cell shape in tissue (Klein and Tebbets 2007). While factors (1−4) are general and also apply to environmental, often extreme habitats, factors (5−8) are more effective under conditions of tissue invasion. Enhancing evasion of the host's immune response is indispensable for fungi with an invasive strategy. In contrast, opportunists lack a strategy that includes tissue invasion; instead, they tolerate extreme environmental conditions that may coincidentally be effective in tolerating the immune response.

Primary pathogens are largely able to evade phagocytosis by macrophages and neutrophils, the infection being resolved later by acquired cellular immunity (Horwath et al. 2015, Fig. 2). Biologically active proteases, beta-glucan and mannans from fungi stimulate innate immune cells, such as dendritic cells, resulting in the production of cytokines (Interleukin-6, IL-12, and IL-23) and proinflammatory mediators (Bartemes and Kita 2018), and subsequently Th1-, Th2-, and Th17-type CD4 T-cells provide protection. For effective control of the pathogen, the correct balance is towards Th1 and Th17 cells and the production of interferon gamma-stimulating phagocytosis (Seyedmousavi et al. 2014). *Talaromyces marneffei* is in the endemic areas of bamboo rats in Southeast Asia even an AIDS-defining fungus (Maniar et al. 2005). In AIDS, the T-helper cells are affected, and therefore patients are less able to control fungal infections that are normally controlled by CD4^+^ immunity; hence the primary pathogens tend to be AIDS-associated (Almeida 2008, Brown et al. 2014, Devi et al. 2020, Myint et al. 2020, Qin et al. 2020; Centers of Disease Control List of AIDS-defining illnesses). In recent years, *T. marneffei* has been emerging in non-AIDS populations (Bai et al. 2021), which suggests that the fungus goes through a process of adaptation; possibly the human becomes a preferred host for this fungus. It should be noted that well-adapted pathogens show low virulence to their preferred host, but may cause severe infections in non-optimal hosts, which makes the distinction of pathogenic *versus* opportunistic behavior less clear. In addition, infections tend to take a more serious course when the host is immunocompromised. The prime control mechanism is delayed, via the acquired, adaptive arm of immunity (Schaffner 1989). The infection kinetics show an initial increase, followed by near-resolution, the fungus residing in dormancy (Brown et al. 2013, Crum 2022) and is reactivated with T-cell impairment, e.g. in the Human Immunodeficiency Virus-positive population.

**Figure 2.**
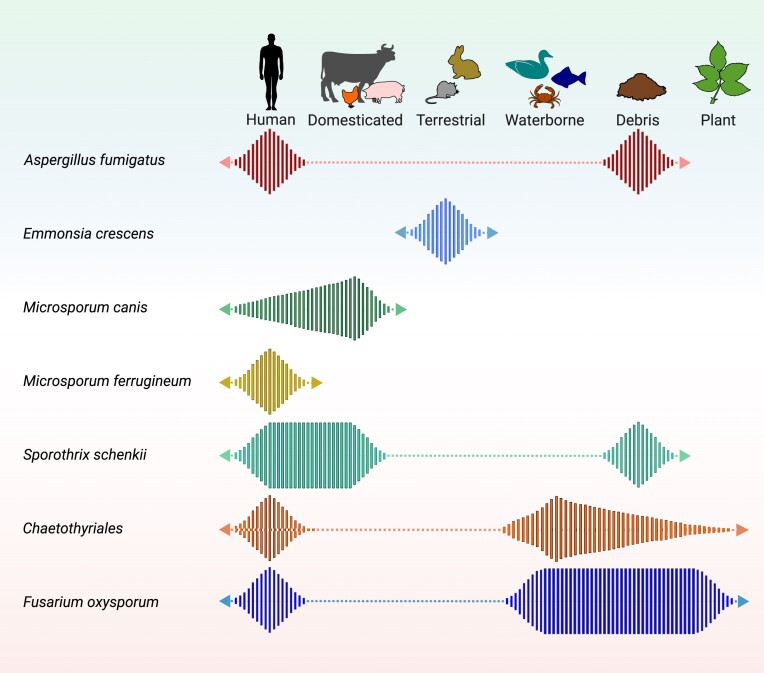
Diagram of preferred habitats (striped areas) of some fungal examples. *Aspergillus fumigatus* on human and plant debris (facultative pathogen), *Emmonsia crescens* with preferred rodent hosts; *Microsporum canis* with preferred feline host but also infecting humans; *Microsporum ferrugineum* with preferred human host; *Sporothrix schenckii* in plant debris but also infecting cats and humans; *Fonsecaea pedrosoi*, probably in environmental debris but also successfully infecting humans; and *Fusarium oxysporum* considered as cross-Kingdom pathogen.

### Examples of opportunistic fungi

Defining primary pathogenicity as beneficial for the species, opportunism in contrast is an infection that may be successful for an individual fungal strain, i.e. the strain is able to survive, but is detrimental to the long-term survival of the species. The infecting strain is lost for a prolonged period or forever for the fungal population, which means suboptimal reproduction and lower than maximum fitness of the species. Vertebrate hosts are an unfavored environment where the fungus struggles to survive. Normally the infection is quickly resolved by inflammation and phagocytosis via macrophages and neutrophils (Loh and Lam 2023). With sufficient and appropriate extremotolerance, however, the fungus may effectively resist, and experience later augmented action via T-cell response. Infection kinetics with the main response by the innate arm of immunity either shows rapid resolution or, if unsuccessful, gradual increase (Schaffner 1989) leading to chronic infection. Antigen recognition via antigen-presenting cells may lead to an inappropriate Th2-cell response with low levels of IFN-γ and high levels IL-4 and IL-10 and insufficient clearance (Seyedmousavi et al. 2014).

A high diversity of factors enhancing an infection has been reported from a wide diversity of fungi. The most devastating infections have been observed with *Mucorales*. Patients with severe infections invariably have significant underlying diseases, such as diabetes, acute myeloid leukemia (AML) or COVID-19 infection, are subjected to immune-lowering therapy, or a combination of these factors (Rudramurthy et al. 2021). Severe, often fatal infections have also been reported from black yeasts and relatives, classified in the ascomycetous order *Chaetothyriales* (Quan et al. 2020). In the past, as with the latter fungi often no underlying disease or immunoincompetence was found in affected patients, several agents were rightfully attributed to the highest biosafety category (BSL-3) in use for fungi. For example, Mitchell et al. (1990) reported fatal dissemination by *Cladophialophora devriesii*, and Tintelnot et al. (1995) by its close relative *Cladophialophora arxii*. Numerous enigmatic severe infections were reported from *Exophiala* species. Dissemination led to a chronic, finally often fatal disease. *Exophiala dermatitidis* was repeatedly responsible for disseminated phaeohyphomycosis (Matsumoto et al. 1993), often in healthy-appearing adolescents (Hiruma et al. 1993) and then sometimes showing neurotropism (Shimazono et al. 1963, Alabaz et al. 2009, Chang et al. 2009). *Exophiala spinifera* showed a similar clinical ability (Dai et al. 1987, Rajendran et al. 2003, Wang et al. 2015), also with some juvenile preponderance, but with a slight osteotropic rather than neurotropic tendency (Li et al. 2011). Of particular interest is *Cladophialophora bantiana*, with about 150 published cerebral cases (Horré and de Hoog 1999, Kantarcioglu et al. 2017), mostly in apparently healthy individuals (Garg et al. 2007, George et al. 2008, Schweizer et al. 2019, Miossec et al. 2020).

However, developments during the last two decades have shed doubt on whether BSL-3 classification was justified for black fungi. Matos et al. (2002) found that the supposed pathogen *Exophiala dermatitidis* to be an abundant and consistent colonizer of the walls in public steam baths and thus would be massively inhaled in countries where no infectious cases by the fungus were known. Similarly, the fungus appeared to have a preponderance in some specific domestic habitats, such as dishwashers (Zalar et al. 2011), railway sleepers (Döğen et al. 2013, Gümral et al. 2014) and other environments that are rich in hydrocarbons (Isola et al. 2013). Analyzing the large number of published cases by de Hoog et al. (2020), the majority of infections occurred in patients with underlying diseases among which were AML (Myoken et al. 2003) and solid organ transplant (Tanuskova et al. 2017). An explanation of the unexplained fatal cases came with the discovery of inherited CARD9-related immunodeficiency (Lanternier et al. 2015). Particularly homozygous CARD9 mutations led to Th17 impairment which increases susceptibility to *Candida*, dermatophytes and melanized fungi (Song et al. 2021). Despite the severe infections caused by members of *Chaetothyriales*, these fungi exhibit ancestral connections with ants (Quan et al. 2020) and lichens (Cometto et al. 2023) but show no trace of vertebrate association. Even the neurotropic species *Cladophialophora bantiana* seems less dangerous than supposed, as it has a possible environmental habitat in sugar-rich plant tissue (Costa et al. 2023).

*Candida auris*, a multidrug-resistant and halo-thermotolerant fungus causes fatal infections mostly in hospitalized patients having a weakened immune system. Later, more isolates were obtained from halophilic environments such as indoor swimming pools, sandy beaches and tidal salt marshes, strains having reduced thermotolerance and drug resistance (Ekowati et al. 2018, Arora et al. 2021, Escandón 2022, Akinbobola et al. 2023). Therefore, it has been hypothesized that the fungus emerged from its natural halophilic reservoir, adapted to endothermic vertebrates as a result of global warming, and was carried by colonized seabirds (Casadevall et al. 2019). Yet, there is no adequate information about the environmental phase of its life cycle or the transmission pathways. The detection of *C. auris* in stored apples in India related to the clinical strains of the fungus, suggested that agricultural use of fungicides can be a selective force for the drug resistance in *C. auris* (Yadav et al. 2022). The fungus can colonize healthy humans and animals without causing any infection (Sexton et al. 2021, Yadav et al. 2023), and during the infection, it is able to escape from the host's innate immune system (Weerasinghe et al. 2023). Karyotype differences and limited recombination (Ross and Lorenz 2020, Wang and Xu 2022) show evidence for the loss of sexuality in *C. auris*.

The fundamentally different immune balance between primary pathogenic and opportunistic fungi was noted in the classical description of infection kinetics by Schaffner (1989). Pathogenicity should not only be described with abiotic and host-related factors, but also with the fungus having a strategy in which a vertebrate host plays a role, anywhere in the lifecycle, enhancing the fitness of the species. This strategy focuses on the evolutionary success of the species, whereas many of the currently described factors to overcome the host immune system are designed for the survival of the individual. In general, host infection of a pathogen increases progeny in the population, whereas in opportunists it is detrimental to the population due to postponed or absence of transmission, and no new generation being founded.

### Examples of fungi between opportunism and primary pathogenicity

**Figure 3.**
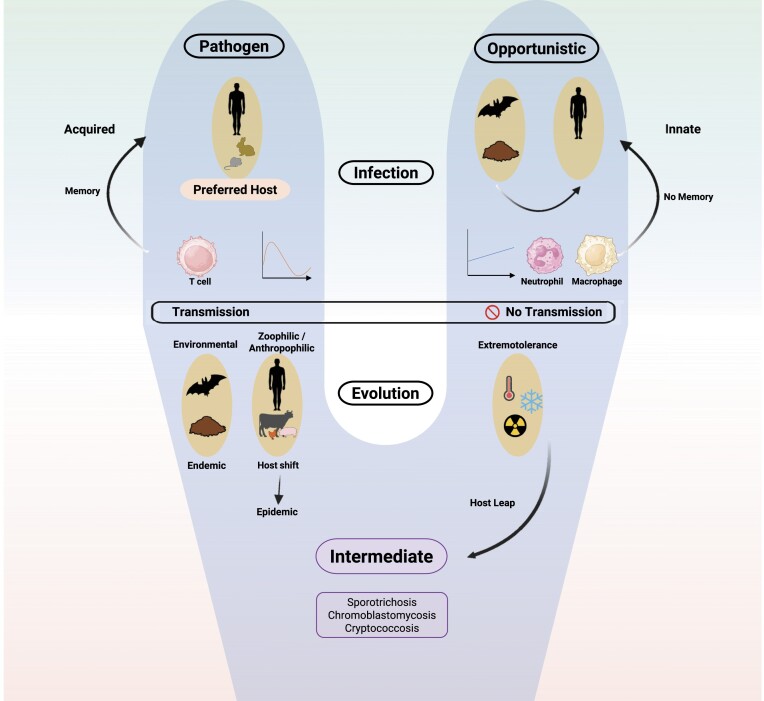
Diagram of fungal ecology with pathogenic and opportunistic arms. Host shifts take place particularly between adjacent hosts, while environmental pathogens are canalized by their abiotic habitat and tend to become endemic. Some opportunistic pathogens with environmental lifestyles survive in animal hosts upon accidental inoculation and might evolve toward primary pathogenicity.

In general terms, the host's immune system recognizes the gradational difference between primary and opportunistic pathogens. The opportunist is usually cleared by inflammation via the innate immune system of macrophages and neutrophils or eosinophils. If the initial response is ineffective, subsequent phagocytosis mediated by CD4 + cells often fails, leading to chronic infection. Infections by host-specific primary pathogens on preferred hosts such as *Histoplasma* are usually suppressed effectively, but with delay and provided that acquired cellular immunity is functional. However, intermediates between primary and opportunistic pathogenicity are numerous, and this approximate bipartition in immune response is not always obvious (Fig. 3). Several fungi, such as *Candida albicans* and *Malassezia* and *Pneumocystis* species naturally colonize hosts as intestinal, cutaneous and pulmonary commensals, mostly without causing fulminant disease. Due to their intimate mammal association they are likely to respond to any breach in the host's immunity.

Cryptococcosis is a disease caused by *Cryptococcus neoformans* and its relative *C. gattii*. The infection is acquired through inhalation, leading to pneumonia, and if not properly treated it may lead to meningitis which can be fatal (Song et al. 2021). *Cryptococcus neoformans* has mostly been regarded as an opportunist causing infection nearly only in immunocompromised patients. The fungus is more common in HIV patients rather than those with a competent innate immune system, and studies have identified mechanisms such as capsule formation, melanin production, titan cell formation, and non-lytic exocytosis that interfere with phagocytosis allowing the expulsion of viable cells from macrophages (Wang et al. 2022). The latter mechanism is related to brain infection and the crossing of the blood–brain barrier, as the fungus can utilize macrophages as “Trojan horses,” allowing yeast cells to migrate into brain tissue (Kronstad et al. 2011, Santiago-Tirado et al. 2017). The fungus has been speculated to have gone through an adaptive leap, from nematodes inhabiting a natural niche of bird guano to human infection (Casadevall et al. 2003). Its close relative *C. gattii* frequently causes deep infection in immunocompetent individuals, which suggests that *Cryptococcus* has shifted from an environmental to a pathogenic lifestyle already long ago.

Ancestral species of the infectious *Sporothrix* clade are environmental, residing in soil and decomposing plant material; some species also associated with bark beetles (de Beer et al. 2016). In the “pathogenic clade” (Zhang et al. 2015), *Sporothrix schenckii* is ancestral; the multiple single-source infections affecting humans typically originated from plant material such as mosses (Dixon et al. 1991), hay (Dooley et al. 1997) or mining wood (Zhang et al. 2015). The emergence of *Sporothrix brasiliensis* in Curitiba since 1990 (Cognialli et al. 2023), originating from Rio de Janeiro (Barros et al. 2004), is notable. Nearly all infections by this species are cat-transmitted (Yeow et al. 2023). It is interesting to look at the disease under a “One Health” perspective, considering that members of the genus affect various mammals (cats, dogs, and humans) increasingly from *S. schenckii* to *S. brasiliensis*. Corrêa-Junior et al. (2023) showed that *S. brasiliensi* virulence factors in cats and humans are similar, indicating minimal adaptation needed between species. It may be assumed that cats while scratching wood and soil, carry *S. brasiliensis* yeast cells under their nails, directly inoculating the invasive form into host tissue (Cabañes 2020). Recently, a suitable type of airborne transmission was noted (Bastos et al. 2022). Thus, the fungus can now transmit between hosts, with the new host becoming preferred. Cases have been described showing that *S. brasiliensis* becomes particularly fulminant in AIDS patients (Poester et al. 2020). Interestingly, the only patient still alive in the report of Cruz et al. (2021) presented IgG antibodies against *Sporothrix*, indicating the presence of immunological memory. *Sporothrix brasiliensis* shows a primary pathogenic profile, unlike its opportunistic ancestor *S. schenckii* (Fig. 1). The dimorphism of *Sporothrix*, where tissue invasion leads to the production of yeast cells rather than hyphae, may be a factor that has enabled this transition.

Chromoblastomycosis (CBM) is a traumatically inoculated skin infection caused by several members of the black yeast order *Chaetothyriales*, such as *Fonsecaea pedrosoi*. Infections should be cleared by inflammatory action of the innate cellular immune system, but insufficient immune response leads to extended acanthotic lesions (Queiroz-Telles et al. 2017). In some *Phialophora* cases, particularly enhanced by genetic immune defects in the Dectin-CARD9 axis (Zhang et al. 2015, Song et al. 2021), the infection becomes chronic due to inappropriate signaling with a Th2 response (Wang et al. 2018), as is also frequently observed in CBM patients (Sobianski Herman et al. 2024). In cutaneous and subcutaneous tissue, the fungus produces large, spherical muriform cells which are resistant to phagocytosis. This form is not unique to the human host but may represent the fungus’ prevalent growth type in tissue, either animal or plant (de Hoog et al. 2007, Fornari et al. 2018). The muriform cell is likely an adaptation to extreme conditions, coincidentally aiding survival in human tissue. In this scenario, the fungus should be able to escape from the infected host, to include the human host as part of a natural life cycle. Chromoblastomycosis is not contagious, and the shedding of skin material loaded with fungal cells into the environment has as yet not been proven.

*Aspergillus fumigatus* is not on this list. The fungus has its natural habitat in self-heating plant debris (Göttlich 1996). It also survives successfully in immunocompromised hosts (Fig. 1). Despite numerous human infections, mammal-to-mammal transmission is absent, and even when this occurs, the few adapted genotypes will soon get lost in the enormous pool of environmental genotypes of the ubiquitous fungus. Azole resistance is an evolutionary driver, but the few human-derived propagules are unlikely to significantly contribute. The fungus evolves under azole pressure in agricultural environments (Verweij et al. 2020), but remains a strict opportunist. Also, some *Fusarium* species commonly invade both plants and humans, for which trans-Kingdom has been suggested (Meza-Menchaca et al. 2020). There are indications that the mechanisms of plant- and human pathogenicity are similar; in that case, these species might be considered facultative pathogens with different hosts (Fig. 1).

## Conclusions and hypotheses

Infectious fungi employ various strategies to infect human hosts. In opportunists, the survival of the species does not depend on the single infecting strain. Transmission drives evolution and adaptation. Without transmission, there is no evolution or host shift, maintaining the fungus-human relationship. At the other end of the spectrum, host-to-host pathogens may shift to a similar, immunologically naïve host, causing an epidemic. Probably the fungus decreases its virulence subsequently, resulting in genotypes that can coexist with the host. Environmental pathogens are less likely to experience host shifts, as they are controlled by their preferred host as well as by the properties of their environmental habitat. Among the environmental fungi, a small number of opportunistic fungi are predisposed to survive in mammalian tissue, potentially leading to pathogenic adaptation. This large host leap from the environment or invertebrates to mammals involves survival mechanisms from their natural habitat.

With respect to the fungi described above, several general statements can be made. Many dermatophyte species have adapted to humans, originating from domesticated animals. This corresponds to the concept of primary host-transmitted pathogens with preferred animal hosts that shift to an adjacent animal host, *H. sapiens*. This can lead to epidemics, as seen with *Trichophyton indotineae* (Gupta et al. 2022). New epidemics are most likely from host shifts involving nearby animal hosts. Possibly the systemic bamboo rat pathogen *T. marneffei* shows signs of adaptation, with a change in its clinical profile (Chan et al. 2016), although still in patients with impaired cellular immunity. Opportunistic mucoralean fungi, despite increased cases during the COVID-19 pandemic (Singh et al. 2021), show no signs of adaptation. Infections result from the emergence of susceptible hosts. Also, black fungi lack adaptation despite frequent chromoblastomycosis in healthy individuals. In this disease, traumatic inoculation is followed by chronic infection due to insufficient clearance of the inoculum. Sporotrichosis seems to be a similar situation: *Sporothrix brasiliensis* cases have surged since 1990, primarily from direct traumatic inoculations by cat scratches (Sanchotene et al. 2015), but these mainly concern direct traumatic inoculations from cat scratches which have dug up the species from the soil. However, the cat-to-human transmission via sneezing (Bastos et al. 2022) suggests human tissue as a new habitat for *Sporothrix*; this is underlined by disseminated sporotrichosis in HIV patients (Poester et al. 2020) and healthy individuals (Fernandes et al. 2018), highlighting the role of acquired cellular immunity in controlling this fungus.

Most fungal pathogens on humans have their origin in non-human mammals and may follow epidemic patterns and adaptations similar to those of viruses and bacteria. However, knowledge of the natural association of fungi with wild animals is limited. Future research on the natural origins of epidemics should include fungi.
